# Wrinkling‐Based Patterning and Recombinant Spider Silk‐Based Coating Technologies ‐ Toward Novel Applications

**DOI:** 10.1002/smll.202508468

**Published:** 2025-12-19

**Authors:** Martin Humenik, Ziwei Zhou, Fabian Kopsch, Andre Knapp, Ziwen Yu, Hendrik Schlicke, Thomas Scheibel, Andreas Fery

**Affiliations:** ^1^ Department of Biomaterials University of Bayreuth Prof.‐Rüdiger‐Bormann‐Str. 1 95447 Bayreuth Germany; ^2^ Leibniz‐Institut für Polymerforschung Dresden e.V. Hohe Str. 6 01069 Dresden Germany; ^3^ Bayreuth Center of Material Science and Engineering (BayMat) University of Bayreuth Universitätsstr. 30 95440 Bayreuth Germany; ^4^ Bavarian Polymer Institute (BPI) University of Bayreuth Universitätsstr. 30 95440 Bayreuth Germany; ^5^ Bayreuth Center of Colloids and Interfaces (BZKG) University of Bayreuth Universitätsstr. 30 95440 Bayreuth Germany; ^6^ Bayreuth Center for Molecular Biosciences (BZMB) University of Bayreuth Universitätsstr. 30 95440 Bayreuth Germany; ^7^ North Bavarian NMR‐Center Universitätsstr. 30 95440 Bayreuth Germany; ^8^ Chair for Physical Chemistry of Polymeric Materials Technische Universität Dresden Mommsenstr. 4 01062 Dresden Germany

**Keywords:** anisotropy, antifouling, bioelectronics, biointerfaces, surface engineering

## Abstract

Wrinkling, formed by stress‐induced energy minimization in thin polymer films, provides a reproducible method for large‐area surface patterning. The resulting nano/micro topographies allow controlled spatial organization of nanomaterials for applications in sensing, optoelectronics, photocatalysis, and soft nanofabrication. The anisotropy of these wrinkled patterns can also be tuned for anti‐biofouling or directional templating of biomolecules, which is crucial for hybrid bio‐interfaces. Complementing this, spider silk‐based surface technologies offer a flexible platform for creating biocompatible and biodegradable coatings. Recombinant spider silk protein technologies enable the modification of intrinsic protein properties (e.g., net charge) or incorporation of new functional elements (e.g., affinity peptides, enzymes). Spider silk‐based coatings have been engineered for antifouling activities or to support cell adhesion and growth. In terms of biomedical applications, enhanced implant performance is feasible as well as tailored tissue engineering approaches. The synergistic combination of wrinkling and recombinant spider silk technology presents exciting opportunities for creation of surfaces with enhanced or new functionalities. For instance, spider silk wrinkled coatings can provide benefit for bioelectronics by encapsulating sensitive biomolecules within a topographically defined matrix, increasing sensitivity and specificity. This approach also offers innovations in biomedical device coatings, tissue engineering platforms (e.g., for neuronal or muscle tissue), large‐scale bio‐selective filtration, and switchable sustainable adhesives.

## Graphical abstract

The synergistic combination of wrinkling and recombinant spider silk technology creates surfaces with enhanced functionalities, unlocking exciting new possibilities. This approach promises innovations in bioelectronics, biomedical device coatings, and tissue engineering, as well as new platforms for large‐scale filtration and sustainable adhesives.

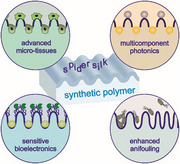

## 1 Wrinkling for Scalable Patterning and Functional Interfaces

Wrinkle structures are a ubiquitous phenomenon in nature, appearing across a wide range of length scales ‐— from meter‐scale patterns in solidified lava flows,^[^ ^1^ ^]^ to micron‐scale features such as human fingerprints, and even sub‐micron wrinkles observed on the surfaces of biological tissues, such as the cornea of zebrafish.^[^ ^2^ ^]^ These patterns originate from mechanical instabilities that arise when thin layers are subjected to external stress. Inspired by these natural examples, researchers have increasingly focused on understanding and harnessing wrinkling as a powerful tool for creating well‐defined, tunable surface topographies in artificial systems. In particular, synthetic polymer films with engineered stiffness gradients provide exceptional versatility for creating such structures, thanks to their tunable mechanical properties, processability, and scalability. The ability to control wrinkle wavelength, amplitude, and orientation opens up new opportunities in diverse technological areas, including optics, electronics, and biointerfaces. Here, while enabling new functionalities and applications itself, the unique topography of polymeric wrinkle structures can furthermore be utilized to pattern hard materials, such as evaporated layers or metal and semiconductor nanoparticles.

### 1.1 Fundamentals of the Wrinkle Formation Process

**Figure 1:**
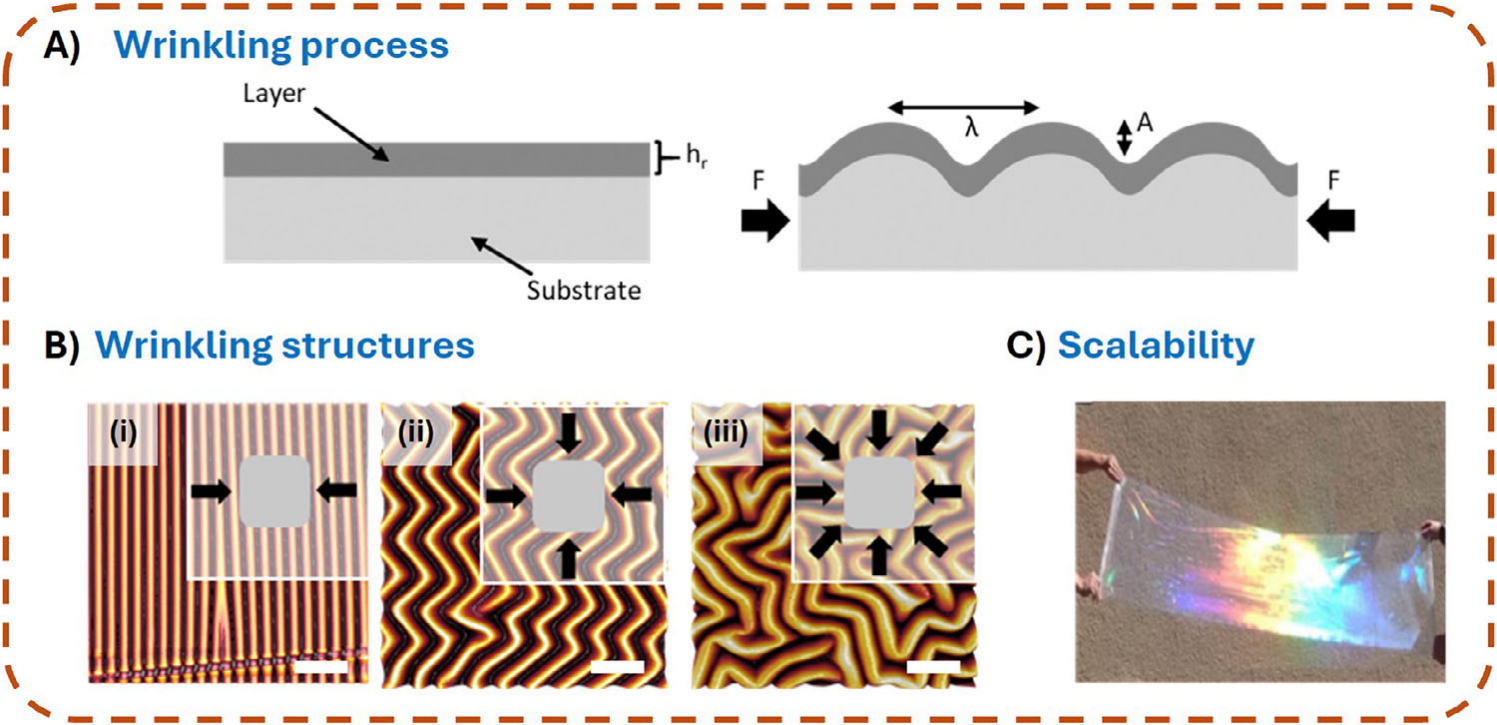
A) Illustration of the wrinkling process in a bi‐layer system composed of a soft substrate and a rigid top layer before compression (left). System under compression by an external force F and the resulting formation of wrinkles with a wavelength λ and structure height A (right). B) Complex wrinkling structures obtained via applying different strain states during plasma treatment of a PDMS substrate and subsequent relaxation (i–iii); Scale bars: 100 µm. C) Example of a meter‐scale film that was nanostructured utilizing the wrinkling process.

In its simplest form, a wrinkling system consists of a bi‐layer, i.e., a thin, stiff top layer covering an elastic substrate material (**Figure** **1**A).

When subjected to in‐plane mechanical compression, the system reaches a critical instability leading to the formation of wave‐like structures in the out‐of‐plane direction. This behavior is driven by the minimization of the system's total energy, where the stiffer top layer undergoes a bending instability.^[^ ^3^, ^4^, ^5^, ^6^ ^]^ The resulting wrinkle structures can be described using two key parameters: wavelength (*λ*) and structure height (*A*). The wavelength represents the distance between two minima or maxima of a wave‐like periodic structure, while the amplitude describes the height difference between a minimum and a maximum within a single period. The wrinkling wavelength can be theoretically predicted through mechanical modeling^[^ ^7^, ^8^, ^9^, ^10^ ^]^ as described in Equation (1).

(1) $$\lambda =2\pi h_{r}{\left(\frac{E_{r}\left(1-v_{s}^{2}\right)}{3E_{s}\left(1-v_{f}^{2}\right)}\right)}^{\left(1/3\right)}$$

where *h*_r_, represents the layer thickness of the stiff layer, and *E*_s_ and *E*_r_ denote the Young's moduli of the substrate and the stiff layer, respectively. The structure height (*A*) can similarly be determined using the critical strain (*ε*_c_), which defines the minimum strain required for wrinkle formation described in Equation (2).

(2) $$A=h_{r}{\left(\frac{\varepsilon }{\varepsilon_{c}}-1\right)}^{1/2}$$

The choice of substrate material is essential for the targeted generation of wrinkle structures. Suitable materials must exhibit high flexibility to accommodate the deformations required for instability while also possessing a nearly linear stress–strain behavior to facilitate accurate predictions of wrinkle morphology. Elastomers, such as polydimethylsiloxane (PDMS), are particularly suitable as they not only exhibit the necessary elasticity but also allow their mechanical properties to be precisely tuned by varying the mixing ratio of their precursors. For PDMS substrates, in situ plasma or ultraviolet/ozone (UV/O_3_) treatment has become the standard method for surface wrinkle structuring. These treatments oxidize and crosslink the substrate near the surface and enable a highly controlled formation of a nanometer‐thin, stiff, glass‐like top layer on PDMS. A key advantage of this method is the seamless coupling between the modified surface and the unmodified bulk PDMS, eliminating the need for additional layers. The thickness and stiffness of the modified layer can be precisely adjusted by selecting appropriate process parameters. In addition to plasma and UV/O_3_ modifications, other techniques can be used to produce a thin, stiff layer. Thin metal films such as gold, silver, platinum, aluminum, or molybdenum can be deposited via sputtering, allowing precise control over layer thickness and homogeneous coverage of the substrate.^[^ ^11^, ^12^, ^13^, ^14^, ^15^, ^16^, ^17^, ^18^ ^]^ Alternatively, thin polymer films such as polystyrene or polytetrafluoroethylene can be used as the top layer, provided they are significantly stiffer than the substrate.^[^ ^19^, ^20^, ^21^, ^22^, ^23^ ^]^ Although these materials may form discrete and homogeneous layers, ensuring proper adhesion between the substrate polymer films remains a challenge. Surface activation techniques, such as plasma treatment or wet‐chemical processes, are often required to improve bonding.

When stretching occurs in multiple directions, more complex, multidimensional wrinkle patterns emerge (Figure 1B). While, for comparison, Figure 1B i depicts the unidirectional wrinkling patterns formed after application of uniaxial strain, Figure 1B ii depicts the formation of a zig‐zag pattern formed upon surface plasma treatment of PDMS under biaxial loading. Furthermore, labyrinth‐like structures can be obtained by applying multiaxial strain during the plasma process (figure part iii). In this case, lacking a preferential strain orientation, the wrinkle structure loses its uniform periodicity.

A key advantage of the wrinkle formation process compared to conventional structuring techniques, such as laser structuring, is its high scalability. This is exemplified by wrinkle formation on PDMS using plasma or UV/O_3_ treatment. Both the plasma dose and the applied mechanical strain allow precise control over the periodicity and structure height. The periodicity can be adjusted within a range of ≈300 nm to over 80 µm,^[^ ^2^, ^24^ ^]^ while the corresponding structure height is typically three to eight times smaller. The maximum processable surface area is dependent on the plasma source. While the use of low‐pressure plasma or a UV/O_3_ chamber limits the structured area to the size of the processing chamber, an atmospheric wrinkle formation process—such as one initiated by a movable plasma jet‐—enables the nanoscale structuring of meter‐scale areas (Figure 1C).

The formation of tailored, wrinkled surfaces on elastomeric materials has a strong application potential in various fields. Commonly, two different approaches are considered: On the one hand, the characteristics of wrinkled surfaces can be directly utilized to influence their interactions with materials or objects.^[^ ^25^, ^26^, ^27^, ^28^, ^29^, ^30^ ^]^ On the other hand, as discussed in the following section, the periodic, highly ordered patterns can be used as templates to guide and arrange matter, such as colloids and molecules.^[^ ^31^, ^32^, ^33^, ^34^, ^35^, ^36^ ^]^

### 1.2 Wrinkle‐Templated (Nanoparticle) Assembly

The generation of well‐defined wrinkle patterns on polymer surfaces, as discussed above, has opened up a versatile platform for template‐assisted assembly of matter on the nano‐and micro‐scale. Wrinkles, produced by controlled mechanical instabilities in thin films, offer a cost‐effective and scalable alternative to conventional lithographic techniques for fabricating ordered nano‐/micro‐material arrays.^[^ ^37^ ^]^

**Figure 2:**
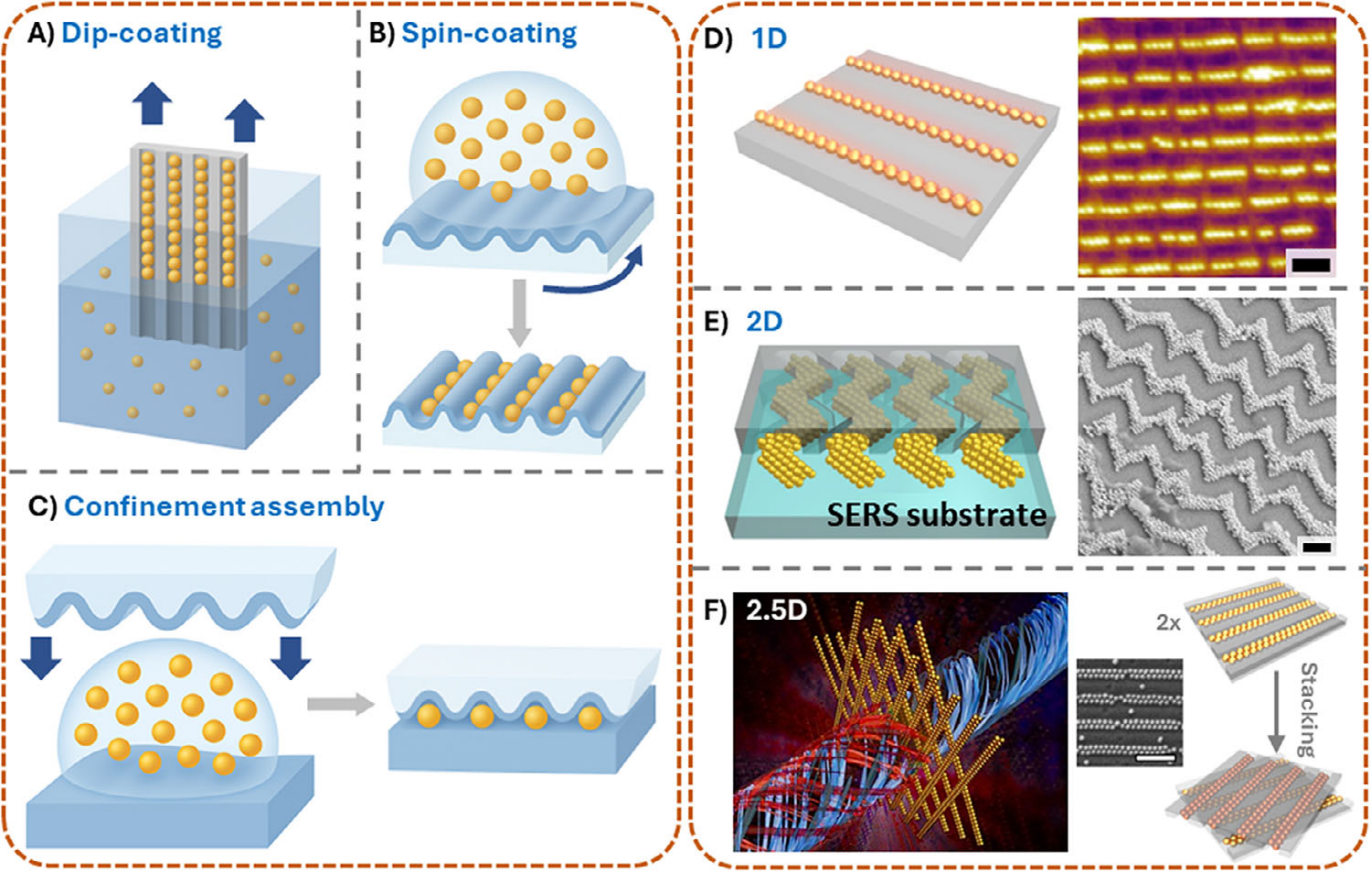
Wrinkled structures as templates for patterning colloidal materials. A) Dip‐coating assembly: Nanoparticles are deposited into wrinkle grooves as a wrinkled substrate is slowly withdrawn from a colloidal suspension. B) Spin‐coating assembly: Centrifugal force and solvent evaporation drive the selective accumulation of nanoparticles along wrinkle valleys during spinning. C) Confinement assembly: Nanoparticles are guided into the wrinkle structure under a confined geometry between the wrinkled PDMS and a flat surface during solvent evaporation. D) 1D assembly: Linear chains of nanoparticles are aligned along uniaxial wrinkle troughs, forming anisotropic nanostructures. Reproduced with permission from Ref.[31] E) 2D assembly: Zigzag wrinkles formed by biaxial stretching guide nanoparticles into multidirectional arrays across the surface. Reproduced with permission from Ref.[40] F) 2.5D assembly: Multilayered or angled stacking of 1D assemblies enables the formation of 2.5D chiral architectures with potential for reconfigurable functional materials. Reproduced with permission from Ref.[41] Scale bars: 500 nm in (D–F).

Wrinkled substrates inherently provide periodic channels and valleys that serve as physical templates to direct the spatial organization of nano‐ and microscale matter. Recent studies have shown that wrinkle‐assisted colloidal self‐assembly can effectively guide nanoparticles into predefined patterns with high spatial precision.^[^ ^32^, ^38^ ^]^ Typically, colloidal particles are deposited from solution onto wrinkled polymer surfaces via several distinct assembly strategies (**Figure** **2**A–C). Taking nanoparticle assembly as an example, dip‐coating assembly^[^ ^34^ ^]^ (Figure 2A) involves vertically immersing and withdrawing the wrinkled PDMS substrate from a colloidal suspension. As the substrate is slowly pulled out, capillary forces at the liquid–air interface promote the selective deposition of nanoparticles into the wrinkle grooves. This method benefits from directional control and is particularly effective for assembling particles along the wrinkle troughs with moderate ordering. Spin‐coating assembly^[^ ^31^ ^]^ (Figure 2B) relies on centrifugal force to distribute nanoparticles over the wrinkled surface. Upon spinning, solvent evaporation and particle flow result in the preferential accumulation of nanoparticles within the wrinkle valleys. While this method enables rapid coating over large areas, the degree of particle alignment can be less controlled compared to dip‐coating. Confinement assembly^[^ ^39^ ^]^ (Figure 2C) employs a top‐down approach in which a droplet of nanoparticle dispersion is sandwiched between the wrinkled PDMS template and a flat substrate. The confined geometry enhances particle packing and guides the nanoparticles into the wrinkle structure during solvent evaporation. This method allows precise spatial confinement and high packing density, making it particularly useful for constructing hierarchical or highly ordered nanostructures. Together, these techniques highlight the versatility of wrinkle‐guided assembly, offering tunable control over nanoparticle positioning, density, and pattern fidelity. The wrinkle topography acts as physical guidance, directing nanomaterials to settle preferentially within valleys or on ridges, depending on the balance between substrate interactions and capillary forces. The resulting assemblies often exhibit a high degree of order, with array spacing precisely defined by the wrinkle dimensions. By tailoring the wrinkle morphology, it is possible to finely tune the arrangement and periodicity of assembled nanostructures. Furthermore, once formed, these assemblies can be transferred onto other substrates using techniques such as contact printing,^[^ ^31^ ^]^ extending their utility for integration into functional devices.

The wrinkle topography offers remarkable versatility in directing the organization of nanomaterials across different spatial dimensions (Figure 2D–F). Using traditional 1D wrinkles,^[^ ^31^ ^]^ nanoparticles can be assembled into linear chain‐like structures aligned along the wrinkle troughs (Figure 2D). These 1D assemblies offer excellent spatial control and are well‐suited for applications such as anisotropic conductive films or optical sensing. By introducing an additional stretching direction during wrinkle fabrication ‐ transitioning from uniaxial to biaxial strain ‐ 2D zigzag wrinkle patterns can be formed.^[^ ^40^ ^]^ These complex surface architectures enable the guidance of nanoparticles into more intricate, planar arrangements with multiple directional alignments (Figure 2E). Such 2D assemblies are particularly attractive for creating plasmonic metasurfaces or hierarchical sensing platforms. Further advancing the structural complexity, 1D nanoparticle chains can be arranged at defined angles relative to one another to create three‐dimensional (2.5D) chiral architectures^[^ ^41^ ^]^ (Figure 2F). This is typically achieved by sequential assembly and re‐orientation steps, enabling the controlled crossing of wrinkle‐guided chains into multilayered structures. Notably, these 2.5D constructs exhibit chiral optical properties and can be reversibly assembled and disassembled with relative ease, offering a simple yet robust route toward reconfigurable nanomaterials with potential applications in chiral sensing, metamaterials, and optoelectronics.

In summary, the use of polymeric wrinkles as templates for nanoparticle assembly represents a promising strategy combining mechanical self‐organization with colloidal assembly principles.^[^ ^35^, ^36^ ^]^ By harnessing the tunable nature of wrinkle patterns, researchers can achieve highly ordered nanostructures with precisely defined inter‐particle distances,^[^ ^32^, ^33^, ^34^ ^]^ paving the way for innovative applications in advanced functional materials.^[^ ^25^, ^35^, ^42^, ^43^ ^]^

### 1.3 Potential Applications of Wrinkle‐Templated Nanomaterials

**Figure 3:**
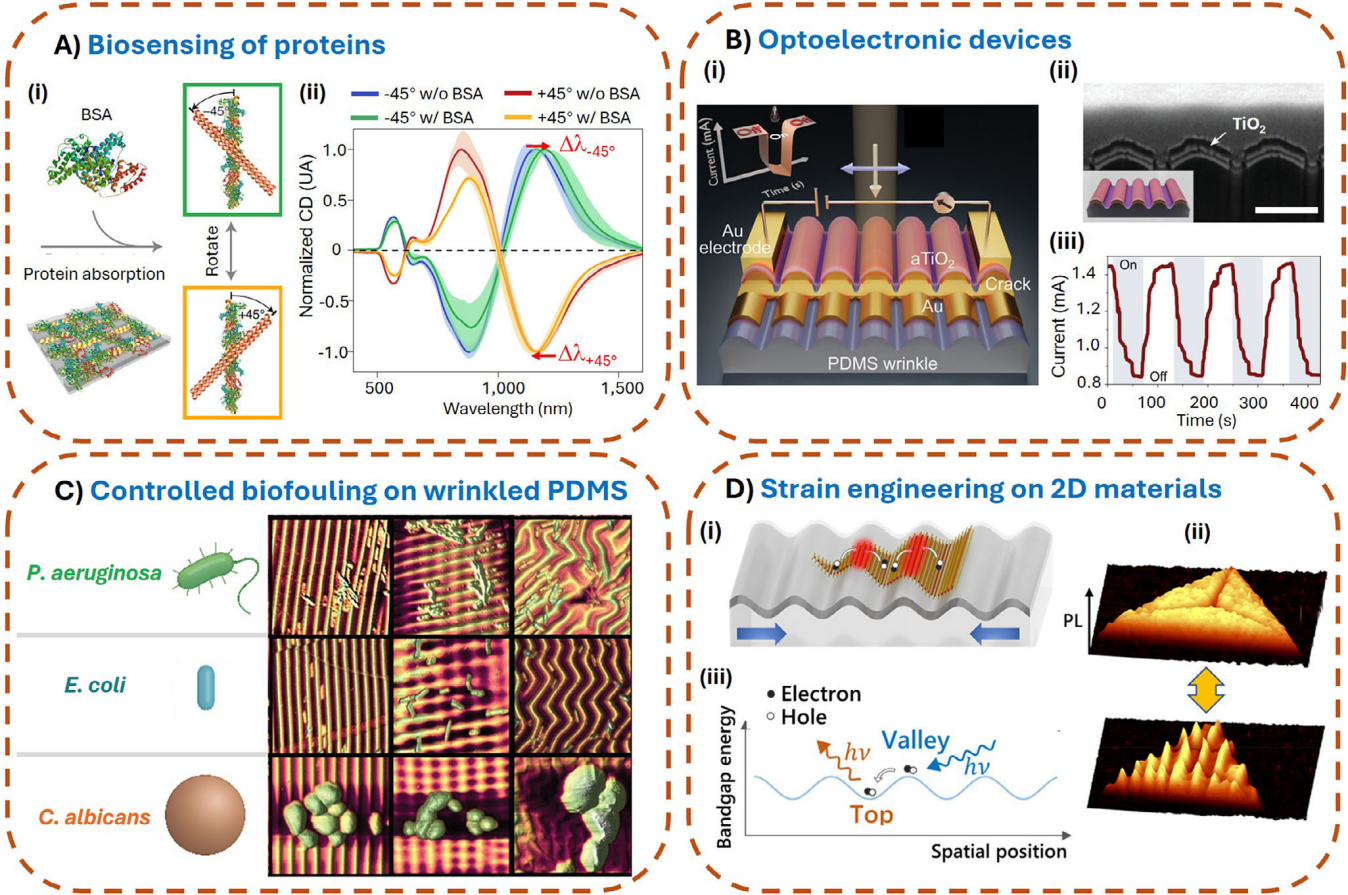
Applications of wrinkled structures in different fields. A) Biosensing of proteins: nanoparticle assembly using wrinkled templates: i) Chiral molecules like bovine serum albumin (BSA) can be detected by using stacked, chiral nanoparticle assemblies. ii) The corresponding normalized CD‐spectra of a −45°/+45° chiral stack before (blue/red, “w/o BSA”) and after BSA coating (green/orange, “w/ BSA”). Reproduced with permission from Ref.[41] B) i, ii) Wrinkled substrates covered with Au and TiO_2_ are used as plasmonic photoresistors. iii) Time‐dependent current signals upon transient light exposure. Scale bar: 500 nm. Reproduced with permission from Ref.[42] C) Wrinkled surfaces for controlling biofouling. Microorganisms of various sizes and shapes were studied on differently wrinkled structures, which resulted in a decrease or increase of settlement depending on the wrinkle morphology. Reproduced with permission from Ref.[45] D) Wrinkled surfaces are used for tuning the bandgap of 2D semiconductor materials. i) Upon depositing 2D materials onto wrinkle structures they follow the wrinkle curvature (ii). The induced deformation results in variations of the materials’ bandgap, which can, e.g., lead to exciton funneling. iii) Laterally resolved photoluminescence intensity of wrinkles under different strain loading. Reproduced with permission from Ref.[48]

The wrinkle templating concept leverages the inherent energy minimization processes during wrinkle formation to create surfaces that are both reproducible and uniform over large areas. This high degree of control in nanomaterial assembly obtained by utilizing wrinkled surfaces is particularly advantageous for applications in optics, electronics, and life science where precise control over the nanomaterial arrangement is essential (**Figure** **3**).

For example, in plasmonic applications, the near‐field coupling between closely spaced metal nanoparticles can be systematically adjusted by varying the wrinkle periodicity, leading to tunable, enhanced localized surface plasmon resonances. This has significant implications for improving biosensing sensitivity once the nanoassembly is chiral (Figure 3A),^[^ ^41^ ^]^ as well as surface‐enhanced Raman spectroscopy (SERS).^[^ ^34^, ^35^ ^]^ Further, the versatility of the technique is underscored by its compatibility with a variety of nanoparticle types ‐ ranging from gold and silver to other functional nanomaterials ‐ thereby broadening its potential applications in optoelectronics, photocatalysis, and beyond.^[^ ^32^, ^33^ ^]^ Due to the unique anisotropy of the wrinkle structures, they act as an efficient shadow mask during glancing angle deposition of metals and semiconductors.^[^ ^42^ ^]^ This enables the formation of desired continuous or discontinuous metallic stripe arrays, which can be further developed into plasmonic photoresistors (Figure 3B).^[^ ^40^, ^42^ ^]^ In addition to these applications, wrinkle‐templated surfaces have shown great promise in the field of anti‐fouling and biointerface control. For instance, bacterial attachment ‐ such as that of *Staphylococcus aureus* or *Escherichia coli ‐* can be effectively reduced or even spatially guided by tuning the wrinkle geometry.^[^ ^44^ ^]^ As shown in Figure 3C, different wrinkle patterns influence the adhesion behavior of *E. coli*, *S. aureus*, *Pseudomonas aeruginosa*, and the fungus *Candida albicans*.^[^ ^45^ ^]^ In particular, recent studies demonstrated that for *P. aeruginosa*, the wrinkle morphology plays a decisive role in significantly decreasing bacterial colonization on PDMS surfaces.^[^ ^45^, ^46^ ^]^ Such topography‐mediated antifouling effects hold great potential for biomedical device coatings and sterile surface design. For anisotropic biomolecular materials such as bacterial cellulose, wrinkles can also serve as guiding templates to align them along the direction of the wrinkle stripes.^[^ ^25^ ^]^ The resulting wrinkle‐based structures exhibit excellent biocompatibility and can further serve as effective substrates for guiding cell alignment.^[^ ^25^, ^47^ ^]^ Another emerging direction is the integration of wrinkles in 2D materials. Wrinkle‐induced mechanical deformation offers a straightforward and scalable route to locally modulate the physical properties of atomically thin layers. For example, controlled bending of 2D semiconductors such as tungsten disulfide can lead to spatial variations in the bandgap, thereby creating strain gradients that funnel excitons into wrinkle valleys. This strategy opens up new opportunities for designing exciton‐based optoelectronic devices with spatially defined functionality^[^ ^48^, ^49^, ^50^, ^51^ ^]^ (Figure 3D).

Wrinkling technologies based on synthetic polymeric systems provide a powerful platform for the controlled fabrication of surface patterns at micro‐ and nanoscale dimensions.^[^ ^29^, ^30^, ^32^ ^]^ This approach synergizes effectively with the customizable functionalization capabilities of nanostructured surfaces made of recombinant spider silk.^[^ ^52^ ^]^ By integrating these two strategies ‐ either by applying wrinkling processes directly onto spider silk layers or by depositing spider silk nanostructures onto pre‐wrinkled substrates ‐ it becomes possible to engineer advanced multifunctional surfaces. To further explore the potential applications enabled by this technological fusion, the following section introduces the properties and functionalization capacity of recombinant spider silk proteins for the design of patterned and bioactive surfaces.

## 2 Functionalization and Patterning of Surfaces Using Recombinant Spider Silk Proteins

### 2.1 Amphiphilic Properties of Spider Silk Proteins

Spider silk fibers outperform most, if not all, natural and synthetic fiber materials in toughness.^[^ ^53^, ^54^ ^]^ The molecular composition of the underlying spider silk proteins (aka spidroins), their nano‐ and mesotructures, as well as their self‐assembly prior to processing into fibers in a sophisticated spinning process, have been well described for major ampullate (MA) silk.^[^ ^55^, ^56^ ^]^ The MA spidroins (MaSps) show a common composition comprising a repetitive core domain (RCD) and non‐repetitive N‐terminal (NTD) and C‐terminal (CTD) domains.^[^ ^57^ ^]^ MaSps are amphiphilic proteins: they contain highly soluble terminal domains, and the RCDs show alternating hydrophobic and hydrophilic patches.^[^ ^58^, ^59^ ^]^ Hence, at high concentrations, MaSps form higher ordered structures necessary for their stabilization in highly concentrated spinning dopes and for achieving multilevel fiber structures^[^ ^54^, ^60^, ^61^, ^62^ ^]^ being decisive for fiber mechanics.^[^ ^63^, ^64^, ^65^ ^]^

Using genetic engineering enables the tailoring of spidroin‐inspired sequences for biotechnological production. The number of repetitive units dictates the molecular weight of a spidroin and can be varied accordingly in recombinant MaSps. Further functionalities, such as affinity peptides or enzymes can be incorporated into the sequences.^[^ ^66^, ^67^, ^68^ ^]^ Processing of spidroins into various morphologies, such as hydrogels,^[^ ^69^ ^]^ particles,^[^ ^70^ ^]^ coatings,^[^ ^71^ ^]^ foams,^[^ ^72^ ^]^ as well as nanofibers^[^ ^73^ ^]^ and nanofibrils^[^ ^74^ ^]^ opens up a multitude of technical and biomedical applications.^[^ ^75^, ^76^, ^77^, ^78^, ^79^, ^80^ ^]^

**Figure 4:**
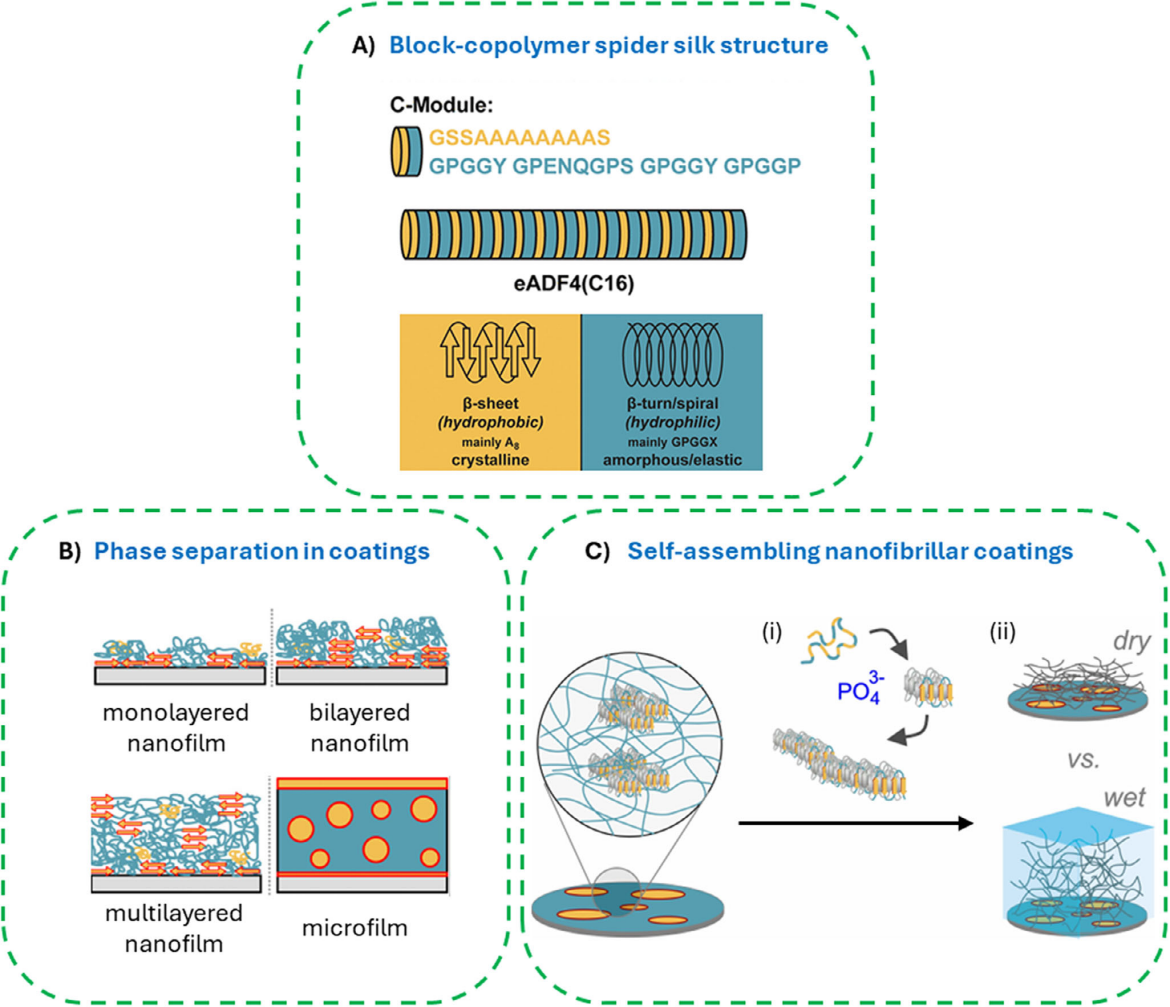
A) Primary structure (amino acid sequence) of an amphiphilic MaSp RCD consensus sequence named C‐module and corresponding secondary structure elements in a respective recombinant MaSp. The two‐color code indicates the block‐copolymer‐like structure where the hydrophobic part of the amino acid sequence is shown in yellow, and the hydrophilic part is shown in blue. B) Nanostructured silk coating ‐ phase separation within the coating depends on the film thickness from the nano‐ to microscale as well as the substrate's surface. Monolayered nanofilms show 2D separation of the hydrophilic and hydrophobic phases. Bilayered nanofilms showed the poly‐Ala‐enriched hydrophobic phase assembled at the silicone surface consequently leading to exposition of the hydrophilic MaSp phase at the protein / air interface. Multilayered nanofilms yield poly‐Ala‐rich surface/protein interfaces and ß‐sheet‐rich protein/air interfaces, which are hydrophobic, and microfilms show microphase separation in the bulk in which ß‐sheet crystallites are embedded as micelle‐like inclusions in an amorphous matrix. They are formed at the protein / air interface as well. Reproduced with permission from Ref.[89] C) Principle of self‐assembling nanofibrillar coatings: immobilized MaSps in a spin‐coated nanofilm reveal ß‐sheet‐rich domains (as shown in the enlargement). These domains i) nucleate self‐assembly of monomeric proteins into surface immobilized fibrils in a process accelerated by phosphate ions. Resulting nanofibrillar coatings ii) exhibit nanohydrogel properties, such as swelling, softening, and low adhesion forces in an aqueous environment. Reproduced with permission from Ref.[92].

The RCDs of MaSps are intrinsically unstructured prior to fiber processing and comprise hydrophobic poly‐Ala motifs (from here on named A‐block) and hydrophilic Gly‐rich regions (B‐block)^[^ ^81^ ^]^ (**Figure** **4**A). Such (AB)_x_ block‐copolymers typically undergo phase separation phenomena either in solution or on hydrophobic/hydrophilic surfaces as well as interfaces, depending on the block‐length ratio and Flory–Huggins interaction parameters.^[^ ^82^, ^83^, ^84^ ^]^ In spidroins, the A‐block forms β‐sheets and stacked crystallites, whereas the B‐block forms random coil and helical conformations linking the β‐sheet regions. One of the best studied MaSps is eADF4(C16), a genetically engineered variant comprising 16 repeats of a consensus sequence (C‐module) of a MaSp (Araneus diadematus fibroin (ADF) 4) from the European garden spider *Araneus diadematus*.^[^ ^66^, ^85^, ^86^, ^87^ ^]^ The C‐module perfectly matches a (AB)x amphiphilic architecture (Figure 4A). The role of the A and B blocks, the number of repetitive units, as well as processing parameters yield nanophase separation and self‐assembly.^[^ ^87^, ^88^, ^89^, ^90^, ^91^ ^]^ Using eADF4(C16), two types of material coatings have been produced: i) film‐type coatings with phase‐separated hydrophobic and hydrophilic blocks (Figure 4B) and ii) self‐assembled immobilized nanofibrillar networks (Figure 4C).

### 2.2 Nanostructured Spider Silk Films

MaSp films can be processed out of organic or aqueous solvents upon deposition on substrates via drop casting, spin‐, spray‐, or dip‐coating. After deposition and drying, the MaSps reveal a high content of random coil structures.^[^ ^85^, ^88^, ^93^, ^94^, ^95^ ^]^ Such films are typically highly soluble in water and hence limited in applications. Film post‐treatment with primary alcohols,^[^ ^85^, ^88^, ^96^ ^]^ heat, or water vapor^[^ ^97^, ^98^ ^]^ is therefore used to increase β‐sheet content and solvent and chemical stability of the films.^[^ ^85^, ^94^ ^]^ The conformational changes mainly occur in the poly‐Ala region,^[^ ^95^, ^97^ ^]^ showing a wide distribution of β‐sheet nanocrystallite sizes from 2 to 40 nm.^[^ ^94^ ^]^ Interestingly, it is possible to axially align the β‐sheets upon film stretching.^[^ ^95^ ^]^

eADF4(Cx) with varying numbers of the C‐module (x = 1, 2, 4, 8, 16) have been used to study phase separation phenomena in films in detail. Peptide‐like assembly versus protein‐like folding behavior was different below and above a sequence length cut‐off of 70 amino acids. In the case of eADF4(C16) (Figure 4D), secondary structure content and surface hydrophilicity of the films increased with a layer thickness of up to 600 nm due to phase separation, orienting the hydrophobic β‐sheet crystalline domains toward the substrate/protein and protein/air interfaces.^[^ ^89^ ^]^ Thicker films (1–2 µm) revealed microphase separation depending on the hydrophobicity of the underlying substrate surface reversing the substrate wettability, i.e., on a highly hydrophobic substrate the surface of the resulting coating appeared more hydrophilic and *vice versa*.^[^ ^89^, ^90^, ^91^, ^99^ ^]^

### 2.3 Self‐Assembled Nanofibrillar Coatings

eADF4(C16) is also one of the best studied examples in the context of nanofibril formation. Triggered by kosmotropic ions, e.g., phosphate ones, the protein self‐assembles into nanofibrils with a cross‐β conformation. The process is nucleation dependent, whereas nuclei not only form in solution. Also, surfaces made of the same protein, such as of MaSp microparticles, chemically immobilized MaSp monolayers, or spin coated MaSp nanolayers can nucleate self‐assembly, enabling the formation of immobilized fibril‐based coatings^[^ ^87^, ^100^, ^101^, ^102^, ^103^ ^]^ (Figure 4C).

Due to the protein's amphiphilic properties, also other substrates, such as glass, stainless steel, silicon, and TiO_2_, could be covered with fibrillar networks.^[^ ^104^ ^]^ Generally, the thickness of nanofibrillar coatings could be adjusted from monolayers up to several hundred nanometers by changing the protein concentration in solution, assembly time, and phosphate concentration. The self‐assembled layers revealed nanohydrogel properties, i.e., swelling, softening, low adhesion forces, and resistance against washing, drying, and wetting (Figure 4C).

### 2.4 Functionalization of Spider Silk Coatings

#### 2.4.1 Functionalization Based of Genetically Modified Spidroins

**Figure 5:**
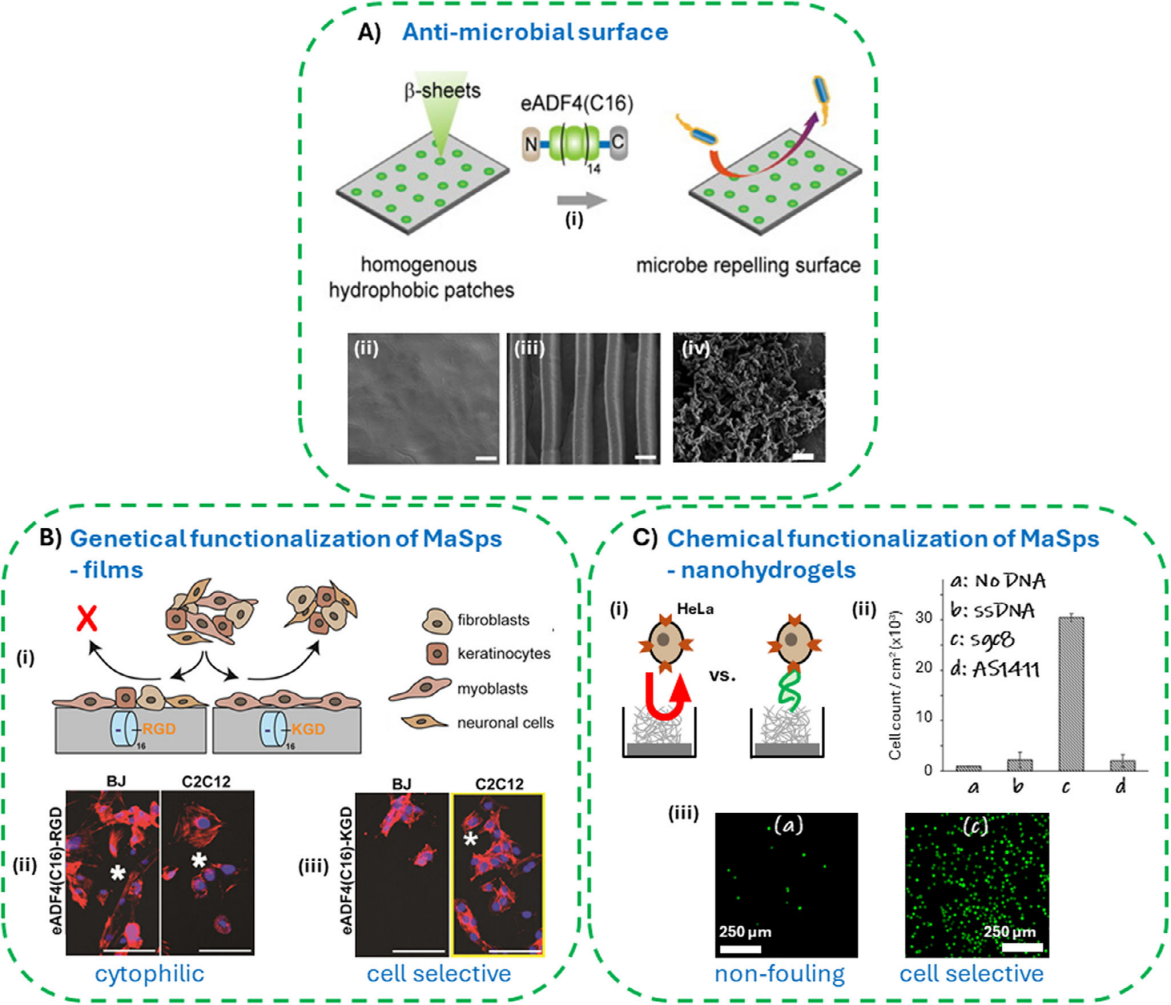
Properties of spider silk coatings. A i) Model illustrating the bacterial repellence of spider silk coatings. Coatings made of the negatively charged eADF4(C16) revealed a homogeneous distribution of nanoscaled hydrophobic patches providing microbe repellence surface characteristics, as demonstrated in case of plain (ii) and structured films (iii) in contrast to polycaprolactone ones (iv); Scale bars: 2 µm. Reproduced with permission from Ref.[113] B i) Schematic of tunable spider silk coatings of tagged eADF4 showing the cytophilic property of an RGD‐variant supporting adhesion and proliferation of a variety of mammalian cell types versus cell selective properties of a respective KGD‐variant supporting mainly myoblast adhesion and proliferation. ii, iii) Fluorescent micrographs of fibroblasts (BJ) and myoblasts (C2C12) on eADF4 surfaces. White asterisks highlight clearly spread cells. Scale bars: 100 µm. Reproduced with permission from Ref.[107] C i) Schematic of cell‐repellent unmodified eADF4(C16) nanohydrogels compared to chemically modified nanohydrogels with DNA‐aptamers, which are capable of cancer cell marker targeting allowing cell‐specific immobilization. ii) Analysis of cell binding on modified nanohydrogels. HeLa cancer were seeded on unmodified (a), arbitrary single stranded (ss) DNA (b), and aptamer‐modified nanohydrogels (c ‐sgc8, d – A1411) and counted after the washing steps showing specific binding of the sgc8 aptamer targeting marker Protein Tyrosine Kinase of HeLa cells. iii) Representative fluorescent micrographs demonstrating non‐fouling properties of unmodified versus sgc8‐aptamer modified eADF4(C16) nanohydrogels. Reproduced with permission from Ref.[120].

The recombinant spider silk technology allows to easily modify the spidroin's sequences, e.g., to exchange all glutamic acid residues in negatively charged eADF4(C16) with glutamine ones to yield the neutral variant eADF4(Ω16) or with lysine ones to yield the positively charged eADF4(κ16).^[^ ^105^ ^]^ Coatings based on the negatively charged and neutral variants displayed significantly reduced adhesion and proliferation of cells, as well as no fibrin formation and no blood coagulation. In contrast, films made of the positively charged MaSp supported interactions with cells and showed enhanced blood coagulation on their surface.^[^ ^105^, ^106^, ^107^, ^108^, ^109^, ^110^, ^111^, ^112^ ^]^ In addition, the coatings made of negatively charged eADF4(C16) also showed microbial repellence, due to nanophase separation of the homogeneously distributed hydrophobic β‐sheet rich domains (**Figure** **5**A). In contrast, uncharged eADF4(Ω16) revealed larger hydrophobic patches, not separated by charge repulsion, which could not inhibit microbial interaction on the coatings. The microbe‐repellence of eADF4(C16) could be transferred onto different surfaces made of polymers, metals, or ceramics, and are not dependent on surface topographies.^[^ ^113^, ^114^, ^115^, ^116^, ^117^ ^]^ The nanofibrillar coatings made of eADF4(C16) also possess antifouling properties, i.e., reduced adhesion of bacteria^[^ ^104^, ^118^ ^]^ and mammalian cells.^[^ ^119^, ^120^ ^]^

In general, most coatings made of recombinant MaSps are biocompatible, nontoxic, biodegradable, and do not trigger an inflammatory response, hence being highly suited for various biomedical applications, as demonstrated, for instance, in the case of silicone implants.^[^ ^109^, ^121^, ^122^ ^]^ The use of negatively charged spider silk coatings shows great promise as protective, anti‐adhesive layers, reducing adverse effects of biofouling typically associated with the use of commercially available biomaterials. This can enhance the performance of indwelling implants and devices that are in contact with biological fluids and tissues.

Genetic functionalization of MaSps with diverse protein tags or enzymes ^[^ ^107^, ^123^, ^124^ ^]^ allows to generate cell‐attractive surfaces (Figure 5B).^[^ ^107^ ^]^ Also, interactions with inorganic materials using explicit peptide tags are possible, such as those derived from bone extracellular matrix proteins^[^ ^125^ ^]^ or affinity sequences allowing for specific binding of TiO_2_ or gold nanoparticles (GNPs)^[^ ^124^ ^]^ Targeted fusion of MaSp sequences significantly expands the portfolio of possible applications, e.g., for tissue regeneration or sustainable optical or energy approaches.

#### 2.4.2 Chemical Functionalization of Spider Silk Surfaces

Chemical functionalization of spider silk surfaces can be obtained through existing Glu‐residues using carbodiimide / N‐hydroxysuccinimide (EDC/NHS) to immobilize enzymes or fluorescent dyes.^[^ ^93^ ^]^ Further, using genetic engineering, a single Cys‐residue has been introduced into an eADF4 variant enabling the site‐specific immobilization of GNPs through redox chemistry (Figure 5C).^[^ ^73^, ^88^, ^124^ ^]^ The single amino‐group on the N‐terminus of eADF4(C16) can also be used for site‐specific modifications allowing click chemistry, as demonstrated with hybrid DNA‐spider silk conjugates.^[^ ^74^, ^92^ ^]^ Thereby, DNA‐addressable binding modalities could be achieved for GNPs^[^ ^74^ ^]^ or even cells.^[^ ^119^ ^]^ Functionalization of nanofibrillar coatings with aptamers, i.e., nucleic acids allowing a target specific binding, enabled for instance enzyme depots which could be released on demand via a structural switch of the aptamer,^[^ ^92^ ^]^ or allowed for cancer‐specific cell immobilization in case of cancer marker targeting.^[^ ^119^ ^]^

#### 2.4.3 Spider Silk Patterned Surfaces

**Figure 6:**
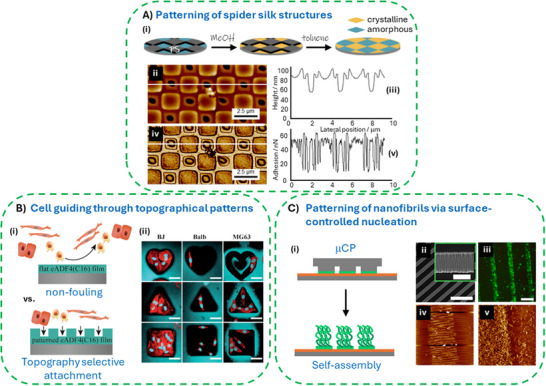
Patterning of spider silk coatings using soft‐lithography techniques. A) Patterned regions could be formed on eADF4(C16) films using capillary transfer lithography. i) Schematic showing the deposition of a polystyrene (PS) mask on an amorphous spider silk film. The unmasked regions transfer into ß‐sheet rich states after MeOH exposure, which leads to patterning after PS mask stripping. The patterns show differences in topography (ii, iii) and adhesion (iv, v). Reproduced with permission from Ref.[130] B) Impact of spider silk coating topography on cell fate. i) Schematic showing non‐fouling properties of a plain surface versus cell selective properties of shape‐defined micro indentations produced using replica molding. ii) Fluorescence micrographs demonstrating accommodation of cells depending on the cell type and the indentation shape (scale bars: 50 µm). Reproduced with permission from Ref.[131] C) Microcontact printing (µCP) of spider silk nanofibrils. i) Schematic of µCP using a PDMS stamp allowing spatially defined deposition of spider silk monolayers and localized growth of fibrils. ii) SEM images of a wrinkled PDMS stamp (scale bars 20 µm). iii) Link^DNA^‐eADF4(C16) conjugate can be locally immobilized on a cap^DNA^ modified substrate using DNA hybridization. The immobilized spidroin locally induces formation of nanofibrils (demonstrated using fluorescently labeled eADF4(C16); Scale bar: 200 µm)). iv, v) Resulting patterns of nanofibrils with sub‐micrometer precision (AFM scans widths are 50 (iv) and 3 µm (v)). Reproduced with permission from Ref.[133]

Micro‐ and nanofabrication technologies are integral to the development of miniaturized systems and devices.^[^ ^126^ ^]^ Using scalable but conceptually simple soft‐lithography methods, high resolution nano‐ and micro‐patterning of protein‐based materials can be achieved.^[^ ^127^, ^128^, ^129^ ^]^ In case of MaSp surfaces, capillary transfer lithography or solvent‐assisted microcontact molding has been used to create polystyrene nanopatterns on eADF4(C16) coatings, which enables highly localized β‐sheet‐transformation of the spider silk in the unmasked areas, resulting in morphological and mechanical sub‐micrometer patterns (**Figure** **6**A).^[^ ^130^ ^]^ Classical poly(dimethylsiloxane) (PDMS) stamps could be used for micromolding in capillaries (MIMIC) or replica molding to organize cells^[^ ^131^, ^132^ ^]^ (Figure 6B) or nanoparticles^[^ ^124^ ^]^ in arbitrary‐shaped topographical patterns. Using PDMS stamps (Figure 6C) in micro‐contact‐printing (µCP) approaches enabled spatially defined immobilization of MaSps, which could be used to nucleate localized nanofibril growth with a sub micrometer resolution.^[^ ^133^ ^]^ Importantly, the micropatterning did not impact the intrinsic antifouling property of eADF4(C16) coatings (Figure 5A).^[^ ^113^ ^]^

In summary, recombinant and genetically engineered MaSps offer biocompatible alternatives to synthetic materials for micro‐ and nanopattern preparations on both biological (soft–wet) and nonbiological (hard–dry) surfaces. Such surfaces have the potential to advance applications, e.g., in bioelectronics, biosensing, and organ‐on‐chip technologies.

## 3 Perspectives for Combining Wrinkling Patterning and Spider Silk Coating Technologies

Wrinkling technology presents a fast, simple, and scalable alternative to traditional soft‐ and photolithography for creating hierarchically patterned surfaces. This method allows for large‐scale production of patterns ideal for the directed organization of nanomaterials^[^ ^134^ ^]^ in applications such as sensing, optoelectronics, and photocatalysis. Integrating wrinkling with recombinant spidroin‐based coatings, renowned for their exceptional biocompatibility, tunable functionality, and environmental stability, significantly expands the potential applications toward a new class of customizable surfaces with functionalities including antifouling properties, affinity binding regimes, and enhanced bio(selective)activity. Two primary strategies can be applied.

**Direct Wrinkling of Spider Silk Layers**: This approach utilizes the inherent stiffness of spidroin films, being similar to those previously made of *Bombyx mori* fibroin, particularly when transformed into a crystalline‐rich state.^[^ ^135^ ^]^ By coating elastic or shape memory polymeric substrates and applying mechanical compression, wrinkling patterns could be directly formed on the spidroin layer. This concept has previously been demonstrated with natural silkworm silk fibroins,^[^ ^136^, ^137^, ^138^, ^139^ ^]^ but recombinant spider silk proteins offer significant advantages due to their controllable composition, a polydispersity index of 1, as well as to the genetic engineering potential within the recombinant production system, in contrast to silkworm fibroins. This could lead to improved reproducibility and higher resolution, in addition to built‐in functionalities of the wrinkled patterns, in case recombinant spider silk proteins are employed in comparison to silkworm silk, which often suffers from higher polydispersity resulting from harsh processing conditions.^[^ ^140^ ^]^

**Spider Silk Coating on Pre‐Wrinkled Substrates**: Alternatively, established synthetic polymeric wrinkling materials can be used to create the desired wrinkled topography first. Recombinant spider silk proteins, which are generally soluble in water and organic solvents, can then be uniformly deposited as nano‐ or micro‐thin coatings onto these pre‐patterned surfaces. A key benefit of this method is that the wrinkling features are precisely controlled by the well‐understood synthetic substrates.

Several critical steps of the coating process must be controlled to obtain uniform spider silk layers, as recently reviewed.^[^ ^52^, ^121^ ^]^ A primary challenge is the potential delamination of spider silk coatings, particularly on flexible polymeric substrates required for wrinkle processing (see Section 1.1). To promote robust integration between the spider silk coating and the wrinkled substrate, surface pretreatments such as plasma activation or silanization can be applied to increase interfacial adhesion. By combining these approaches with the use of further engineered spider silk variants, a broad range of materials^[^ ^108^ ^]^ can be effectively coated with spider silk proteins. These include synthetic polymers such as polystyrene, silicone, polytetrafluoroethylene, polydimethylsiloxane, and polyurethane, as well as metals, glass, silica, and mica.^[^ ^52^, ^121^ ^]^ Moreover, spider silk coatings can also be applied to soft biological materials, including silkworm silk fibroin scaffolds, commercial silk sutures (e.g., Perma‐Hand), and even wood.^[^ ^52^, ^121^ ^]^

Another major challenge lies in achieving a uniform coating on wrinkled substrates. Nevertheless, recent studies have demonstrated that surface‐structured substrates with contours, internal architectures, or complex 3D topographies can be uniformly coated with spider silk using methods such as casting, spin‐coating, spraying, or combinations thereof. ^[^ ^110^, ^113^, ^141^, ^142^, ^143^ ^]^ Notably, effects of substrate texture on the secondary structure and molecular orientation of spider silk were observed in nanofilms of spider silk proteins on nanopatterned surfaces.^[^ ^89^, ^144^ ^]^ The application‐relevant thicker coatings formed on micropatterns with wrinkle‐typical micrometer dimensions did not differ significantly from those on flat substrates.^[^ ^144^ ^]^ Importantly, by designing appropriate spidroin variants, one can tailor functionality of the composite surface, such as antifouling, cytophilic, or cell specific biointeractions.^[^ ^52^, ^121^ ^]^

The complementary strengths of synthetic polymer wrinkling systems and spider silk–based coatings are summarized in **Table** **1**, and their interplay enables the design of multifunctional surfaces with strong potential for novel applications.

**Table 1:** Comparison of synthetic polymer‐based wrinkling systems and spider silk‐based coatings, highlighting their respective strengths and how their combination enables complementary functionalities.

Aspect	Synthetic Polymer Wrinkling Systems	Spider Silk‐Based Coatings	Complementarity / Synergy
Scalability & Patterning	highly scalable cost‐effective tunable wrinkle wavelength (300 nm–80 µm) reproducible over large areas	accessible scalable wrinkled topographies	precise and scalable wrinkle templates with biofunctionality originating from the engineered spider silk sequence
Biocompatibility	biologically inert non‐toxic, limited inherent bioactivity	naturally biocompatible biodegradable non‐toxic non‐inflammatory bioselective tunable degradation rates	wrinkles providing topographic cues for cells engineered spider silk chemistry controls biological signaling
Functionalization	templating nanoparticle assembly modulating bandgaps in 2D materials controlling biofouling	accessibility for diverse genetical and chemical modifications	multifunctional, bioactive and, e.g., optoelectronic surfaces
Physical and Mechanical Properties	engineering of stiffness gradients flexible topographies	high toughness amphiphilicity defined surface net charge	mechanically stable yet bioresponsive surfaces strain‐driven delivery or activation of embedded biofunctional components.

Spider silk coatings, however, can present certain drawbacks, depending on the chosen application. For instance, the swelling and softening of nanohydrogel‐based coatings in aqueous environments^[^ ^92^, ^104^ ^]^ might be detrimental when long‐term stability of wrinkled surfaces is required. At least in biological environments, spider silk film‐based coatings have demonstrated remarkable stability, as shown in the case of spider silk coated silicon breast implants subjected to prolonged *in vivo* testing for 12 months.^[^ ^109^, ^145^ ^]^ Nevertheless, to address potential stability challenges, different strategies could be employed: i) applying mild, site‐selective covalent crosslinking (e.g., using genipin, thiol chemistry, *etc*.)^[^ ^146^, ^147^ ^]^ to restrict chain mobility of the underlying spider silk proteins; and ii) covalently anchoring the spider silk proteins to the wrinkled substrate after plasma activation or silanization to enhance interfacial adhesion and to prevent delamination.^[^ ^121^ ^]^ The effectiveness of these measures should be systematically evaluated through tests of swelling behavior, enzymatic degradation, adhesion strength, and flow stability under relevant conditions for each application to ensure long‐term durability without compromising the tailored biofunctionality of spider silk.

From the synergies presented in Table 1, we envisage several putative low‐ to high‐tech applications. For example, smart adhesives and anti‐adhesives could build on wrinkle‐driven switchable adhesion, complemented by spider silk's tunable antifouling or bioselective properties. Likewise, advanced biosensors could benefit from wrinkle‐guided nanoparticle alignment and enlarged surface area, which ‐ combined with spider silk's capacity to immobilize enzymes, antibodies, or aptamers ‐ could improve sensitivity and stability in complex biological environments. Finally, in case of cell culture systems (such as organ‐on‐a‐chip approaches, *etc*.), wrinkled topographies could mimic extracellular matrix structures and influence mechanotransduction, while spider silk coatings could be engineered with adhesion peptides or growth factor–binding domains to guide cell differentiation and tissue formation.

**Smart Adhesives and Anti‐Adhesives**: Wrinkled spider silk surfaces could be designed to exhibit switchable adhesion properties. By applying a stimulus (e.g., mechanical strain, light, humidity),^[^ ^148^, ^149^, ^150^ ^]^ the wrinkle pattern could change, altering the contact area, and, thus, the adhesion force.^[^ ^151^ ^]^ This could lead to reusable adhesives or surfaces with controlled release properties. The topographical structuring could also be used for direction‐dependent adhesion or self‐aligning surfaces,^[^ ^152^ ^]^ without the need for additional chemical crosslinking, since two wrinkled surfaces are expected to show stronger adhesion when the orientation and periodicity of the wrinkles are matching on both surfaces. Conversely, engineering wrinkles’ nontopographic features combined with antifouling spider silk properties could create highly effective anti‐fouling surfaces enhancing performance of implants and catheters.

**Advanced Biosensors**: Micro‐ and nanotechnologies are playing a central role in advancing the field because of their potential to address challenges in sensitivity, miniaturization, and cost‐effectiveness.^[^ ^153^, ^154^, ^155^, ^156^ ^]^ The wrinkled topography of spidroin‐based coatings could significantly enhance surface area, crucial for immobilizing bioreporters like enzymes, antibodies, or aptamers, which can be genetically fused (enzymes) or chemically coupled (antibodies and aptamers) on the spidroin coating, or integrated into a self‐assembled spider silk nanohydrogel matrix on top. This increased surface area, combined with the spidroin's inherent biocompatibility and anti‐fouling properties, could enable more sensitive and stable biosensors for detecting specific analytes in biological fluids. Furthermore, the wrinkles could potentially serve as templates for aligned nanoparticle arrays, amplifying analyte‐generated signals (e.g., optical, electrical). Overall, this integrated approach would be poised to improve biosensor sensitivity and selectivity within biological environments.

**Cell Culture Substrates**: Biological functions at the cellular and tissue level are rarely governed by a single type of signal. Physical signals, such as surface topography, stiffness, and mechanical strain, influence cytoskeletal organization, cell morphology, and mechanotransduction pathways. These physical cues are particularly valuable for applications in neuronal, cardiac, and vascular tissue engineering.^[^ ^138^, ^157^, ^158^, ^159^, ^160^ ^]^ In contrast, biochemical signals, such as adhesive peptides, growth factors, or antifouling domains ‐ as exemplified on spider silk coatings^[^ ^52^, ^121^ ^]^ ‐ engage receptor‐mediated pathways that regulate gene expression, cell proliferation, and cell differentiation.^[^ ^161^, ^162^, ^163^ ^]^ When combined, these signals act synergistically: the physical architecture provides spatial and mechanical guidance, while biochemical cues deliver molecular specificity. Together, they create a more physiologically relevant microenvironment, enabling precise control over cell fate and function that neither signal alone could achieve.

Moreover, spidroins could be engineered to control the degradation of a coating at rates aligning with tissue formation, which allows the generation of smart surfaces releasing ready‐to‐implant cells sheets.^[^ ^164^ ^]^ Hence, the combination of wrinkled topography and bioactive spidroin coating will enable a synergistic platform: topography influences mechanotransduction, while spidroin bio‐chemistry influences biological signaling.

**Spatially Arranged Multiple Functionalities on Large Areas**: Beyond genetic or chemical engineering, spider silk's inherent ability to transition between amorphous and β‐sheet‐rich structures offers a powerful route to introduce hierarchical patterns and diverse functionalities on large areas.^[^ ^52^ ^]^ Water molecules act as plasticizers in silk coatings, relieving internal strain within the hydrogen‐bond networks.^[^ ^165^ ^]^ This property could be harnessed through selective, large‐scale condensation of solvent vapors into wrinkle valleys.^[^ ^166^ ^]^ For amorphous spider silk films, water condensation can possibly lead to localized dissolution, allowing for spatially controlled deprotection of the underlying polymer substrate. This approach will enable the creation of multi‐functionality surfaces, where the polymer substrate is exposed in the valleys, and the spider silk coating remains on the peaks. Such a dual‐patterned surface could, for example, direct the selective deposition of one type of nanoparticle onto the exposed polymer regions, while affinity‐peptide‐functionalized spider silk binds a second type of nanoparticle, resulting in large‐scale surfaces with unique opto‐electronic properties. Furthermore, if both the spider silk and polymer substrates could be modified with different cell‐binding ligands, this system could facilitate the spatial organization and alignment of two distinct cell types, paving the way for advanced patterned tissue engineering.

**Patterning Spider Silk with Wrinkled Surfaces**: Wrinkled surfaces, characterized by their inherently periodic and anisotropic topographies, could present a compelling platform for guiding the localized deposition of spidroins. Techniques such as MIMIC or µCP, recently demonstrated with small PDMS stamps in proof‐of‐concept studies,^[^ ^132^, ^133^ ^]^ could be applicable to scalable wrinkled substrates. This will allow for the creation of functionalized spidroin patterns ‐ ranging from monolayers to self‐assembled nanofibrillar nanohydrogels ‐ across large surface areas. Furthermore, by tuning the wrinkle wavelength or type (see Figure 1), gradient or hierarchical patterning could be achievable. This combination of wrinkled surface engineering and precise spidroin deposition will unlock significant potential for applications in diverse fields, including tissue engineering and bioelectronics.

In summary, the integration of synthetic wrinkle patterning and recombinant spider silk coatings creates a versatile platform that merges engineered structural precision with biological adaptability, opening pathways for next‐generation applications, e.g., in tissue engineering, bioelectronics, biosensing, and sustainable materials.

## Conflict of Interest

Thomas Scheibel is co‐founder and shareholder of AMSilk GmbH. All other authors declare no conflict of interest.
